# Genomic Characterization and Taxonomic Classification of *Mycobacterium shunyiense* sp. nov., a Novel Nontuberculous Mycobacterial Species from a Human Respiratory Specimen

**DOI:** 10.3390/ijms27156890

**Published:** 2026-08-01

**Authors:** Yang Deng, Xin-Yu Yang, Nen-Han Wang, Bo Li, Hong-Hao Miao, Jie Zhang, Meng-Di Pang, Yi-Xuan Ren, Jun-Li Yi, Man Cai, Jie Li

**Affiliations:** 1 Beijing Center for Disease Prevention and Control, Beijing 100013, China; 2 School of Public Health, Capital Medical University, Beijing 100069, China; 3 Institute of Microbiology, China General Microbiological Culture Collection Center, Chinese Academy of Sciences, Beijing 100101, China

**Keywords:** *Mycobacterium shunyiense* sp. nov., non-tuberculous mycobacteria, genome-based taxonomy, antimicrobial resistance, virulence factors, whole-genome sequencing, polyphasic taxonomy

## Abstract

Non-tuberculous mycobacteria (NTM) are increasingly recognized as opportunistic pathogens, yet accurate species identification remains challenging. In this study, two clinical isolates, 19-857^T^ and 20-525, obtained from patients with suspected pulmonary tuberculosis in Beijing, were characterized using a polyphasic approach integrating phenotypic, biochemical, chemotaxonomic, and genomic analyses. Phylogenetic analysis based on 16S rRNA gene sequences placed both strains within the genus *Mycobacterium*, with highest similarity to *M. peregrinum* DSM 43271^T^ and *M. arcueilense* DSM 46715^T^. However, whole-genome comparisons via average nucleotide identity (ANI) and digital DNA–DNA hybridization (dDDH) yielded values of 85.8–94.1% and 29.1–54.3%, respectively, against their closest relatives—well below the species delineation thresholds (95–96% for ANI; 70% for dDDH)—while the two isolates shared 98.8% ANI and 90.1% dDDH, confirming their co-assignment to a single novel species. Genomic analysis further revealed a repertoire of genes potentially associated with virulence, including glycopeptidolipid biosynthesis, *mce* operons, ESX secretion systems, and mycobactin synthesis, alongside antimicrobial resistance determinants against rifampicin, tetracyclines, and *β*-lactams. Mobile genetic element analysis also uncovered extensive insertion sequences and transposon-related elements with differential distribution between the two strains. Based on the combined evidence, we propose *Mycobacterium shunyiense* sp. nov., with strain 19-857^T^ (=CGMCC 38906^T^) as the type strain. This discovery expands the understanding of NTM diversity and provides genomic reference data for accurate clinical identification.

## 1. Introduction

The formal establishment of the genus *Mycobacterium* occurred in 1896, within the family *Mycobacteriaceae*, where it was initially represented by only *Mycobacterium leprae* and *Mycobacterium tuberculosis* [1]. Among the earliest recognized non-tuberculous mycobacteria (NTM) were saprophytic organisms such as *Mycobacterium smegmatis* and *Mycobacterium phlei* [2]. Although the etiological agent of avian tuberculosis was identified during the late 19th century, its formal designation as *Mycobacterium avium* by Chester did not take place until 1901 [3]. Following this, several additional mycobacterial species linked to animal diseases emerged, including *Mycobacterium lepraemurium* [4] and *Mycobacterium paratuberculosis*. Prior to 1950, only a limited number of other mycobacteria had been documented beyond those already mentioned; among them were *Mycobacterium chelonae* [5], *Mycobacterium marinum* [6], and *Mycobacterium fortuitum* [7].

Since then, the genus has since expanded to 220 validly published species (LPSN, https://lpsn.dsmz.de/ accessed on 1 June 2026), most of which are NTM. Notably, a recent phylogenomic study by Gupta et al. [8] proposed a major reclassification of the genus *Mycobacterium* into an emended genus *Mycobacterium* and four novel genera, including *Mycolicibacterium*, based on comparative genomic analyses. Under this proposed framework, several rapid-growing NTM species—including *M. peregrinum* and *M. arcueilense*—would be reclassified as *Mycolicibacterium peregrinum* and *Mycolicibacterium arcueilense*, respectively. Given the close phylogenetic relationship of our novel strains to these two species, *M. shunyiense* sp. nov. would likely also fall within the proposed genus *Mycolicibacterium* under the Gupta et al. [8] classification. However, as the reclassification of the entire genus *Mycobacterium* remains a subject of ongoing debate and has not been universally adopted in clinical and taxonomic practice, we have followed the traditional genus designation in the present study to maintain consistency with the existing clinical literature and diagnostic frameworks. These organisms constitute a category of actinobacteria that are extensively distributed throughout natural ecosystems, showing marked prevalence in soil, freshwater habitats, and potable water systems [9]. Most members of the genus are saprophytic and pose little threat to human or animal health; however, a minority can trigger opportunistic infections under certain circumstances, especially in hosts with compromised immunity. At-risk groups include patients on chemotherapeutic regimens, individuals with HIV/AIDS, recipients of immunosuppressive therapies following organ transplantation, and those with chronic lung conditions such as chronic obstructive pulmonary disease (COPD), bronchiectasis, or cystic fibrosis [10]. Mycobacteria of the NTM group are highly diverse in their geographic dispersal, phylogenetic placement, and genome dimensions and are typically divided into slow-growing (SGM) and rapid-growing (RGM) categories according to their replication rate [11]. This differentiation hinges on whether visible colonies from a dilute inoculum emerge within a seven-day period. Although the creation of a separate subgenus, *Mycomycobacterium*, for the rapid growers has been advocated by some researchers [12], subsequent studies based on polyphasic taxonomic characteristics—including DNA-DNA hybridization, phage susceptibility, and lipid composition—have demonstrated that the two groups are highly homologous at both genetic and biochemical levels, which does not sufficiently support the establishment of a distinct subgenus [13].

The worldwide burden of diseases attributable to NTM continues to climb, posing a growing public health concern. Among the rapid growers, the *Mycobacterium abscessus* complex (MABC), in particular, has garnered attention as a clinically relevant pathogen and is increasingly recognized as a significant threat to individuals with cystic fibrosis (CF). Effective antimicrobial therapy for MABC infections may span two years or more, and therapeutic failure is frequently accompanied by accelerated deterioration of pulmonary function. Compounding this challenge, the organism displays inherent tolerance to numerous antimicrobial agents, driven both by its intrinsic biological features and by acquired resistance mechanisms, which complicates the clinical management of MABC pulmonary disease [14]. Among the clinically significant NTM, the *Mycobacterium avium* complex (MAC) remains the most common cause of NTM pulmonary disease worldwide, particularly in immunocompetent individuals with underlying lung conditions such as COPD and bronchiectasis. Another important category within the NTM classification system is the *Mycobacterium simiae* complex. Several members of this complex are associated with a spectrum of clinical manifestations, including pulmonary infections and skin and soft tissue lesions, as well as disseminated disease [15].

In this study, two rapidly growing, non-pigmented NTM strains (19-857^T^ and 20-525) were isolated from patients with suspected pulmonary tuberculosis in Beijing. Through comprehensive phenotypic, biochemical, and genomic profiling, we formally propose the recognition of these two strains as representatives of a novel species, for which the name is *Mycobacterium shunyiense* sp. nov., with strain 19-857^T^ designated as the type strain. Genomic exploration further uncovered key genetic elements potentially linked to virulence, antimicrobial resistance, and horizontal gene transfer, offering molecular-level clues to their evolutionary history and pathogenic capacity.

## 2. Results

### 2.1. Physiological and Morphological Characteristics

Morphological analyses observations revealed that the strains 19-857^T^ and 20-525 were Gram-stain-positive, rod-shaped, and nonspore-forming bacteria. When grown on TSA at 37 °C, cells measured approximately 1.32 ± 0.18 μm in length and 0.65 ± 0.05 μm in diameter under electron microscopy (Figure S1). Colonies of strains 19-857^T^ and 20-525 grown on TSA medium and incubated for 5 d were orange, smooth and moist in appearance. Growth was observed after 5 days, with an optimum at 28 °C; good growth also occurred at 25, 30, and 37 °C, whereas only scant growth was detected at 40 °C. Strain 19-857^T^ grew within a pH range of 6.0–8.0 (optimal pH 7.0), while strain 20-525 exhibited a broader pH tolerance (6.0–9.0, with an optimum at pH 7.0). Regarding salt tolerance, strain 19-857^T^ failed to grow at 6.0% NaCl, whereas strain 20-525 only ceased growth at a higher concentration of 7.0% NaCl. Following 5 days at 28 °C, both strains exhibited robust growth on L-J, MB7H10, GYM, and TSA plates, moderate growth on NA agar, and weak growth on R2A. Both isolates were catalase-positive but negative for oxidase, gelatin hydrolysis, and H_2_S production. Both strains exhibited positive enzymatic activities for acid phosphatase, cystine arylamidase, esterase (C4), esterase lipase (C8), leucine arylamidase, lipase (C14) and *β*-glucosidase, based on API ZYM strip tests. Acid production from fermentation was not observed for either strain with the following substrates: amidon, amygdalin, arbutin, D-arabinose, D-arabitol, D-ardonitol, D-cellobiose, D-fucose, D-galactose, D-glucose, D-lactose, D-lyxose, D-maltose, D-mannose, D-melezitose, D-melibiose, D-raffinose, D-ribose, D-saccharose, D-sorbitol, D-tagatose, D-trehalose, D-turanose, dulcitol, D-xylose, erythritol, gentiobiose, glycogen, inositol, inulin, L-arabinose, L-arabitol, L-fucose, L-rhamnose, L-sorbose, L-xylose, Mehyl-*α*-D-glucopyranoside, Mehyl-*α*-D-mannopyranoside, Mehyl-*β*-D-xylopyranoside, N-acetylglucosamine, potassium 5-ketogluconate, salicin and xylitol. In contrast, both strains produced acid from a limited subset of substrates, including D-fructose, D-mannitol, esculin ferric citrate and potassium gluconate. Detailed biochemical characteristics of strains 19-857^T^ and 20-525, together with those of the reference strains *M. peregrinum* DSM 43271^T^ and *M. arcueilense* DSM 46715^T^, were summarized in Table 1.

### 2.2. Drug Susceptibility and Resistance Patterns

The two test isolates displayed comparable antimicrobial susceptibility profiles. Both strains 19-857^T^ and 20-525 were susceptible to amikacin, cefoxitin, ciprofloxacin, clarithromycin, imipenem, linezolid, moxifloxacin, tigecycline, meropenem, tobramycin, trimethoprim/sulfamethoxazole, and rifabutin. In contrast, resistance was observed against amoxicillin/clavulanate, rifampicin, minocycline, and doxycycline (Table S2).

### 2.3. Chemotaxonomic Properties of Strains

The chemotaxonomic characteristics of strains 19-857^T^ and 20-525 were consistent with their assignment to the genus *Mycobacterium*. Strains 19-857^T^ and 20-525 shared major fatty acid components—myristic acid (C_14:0_) and oleic acid (C_18:1_*ω9c*, sum feature 3 (C_16:1_*ω7c*/C_16:1_*ω6c*))—each accounting for over 10% of the total fatty acid content, a profile typical of *Mycobacterium* species (Table S1); in contrast, minor quantities (1.5% and 1.8%) of *anteiso*-C_17:0_ and C_16:1_*ω11c* were uniquely present in strain 19-857^T^, while being absent from strain 20-525 and the two close relatives (*M. peregrinum* DSM 43271^T^ and *M. arcueilense* DSM 46715^T^). The polar lipid profiles of the two isolates were largely similar, both containing diphosphatidylglycerol (DPG), phosphatidylethanolamine (PE), phosphatidylinositol (PI) and phosphatidylinositol mannosides (PIM). However, an additional glycolipid (GL) was identified in strain 20-525 (Figure S2). Analysis of whole-organism hydrolysates revealed the presence of *meso*-diaminopimelic acid (*meso*-A_2_pm) in both strains 19-857^T^ and 20-525, with MK-9(H_2_) serving as the predominant menaquinone.

### 2.4. Physiological Characteristics and Genome Analysis Overview

The nearly complete 16S rRNA gene sequences of strains 19-857^T^ and 20-525 were determined, each comprising 1482 bp, with GenBank accession numbers PZ514122 and PZ514123, respectively. Sequence comparison between the two isolates showed 99.6% identity. BLAST analysis (BLAST+ 2.13.0) against the GenBank database revealed that strain 19-857^T^ shared 99.6% similarity with both *M. peregrinum* DSM 43271^T^ and *M. arcueilense* DSM 46715^T^, followed by *M. septicum* DSM 44393^T^ (99.3%) and *M. nivoides* JCM 32796^T^ (99.3%). Strain 20-525, by contrast, exhibited 100.0% similarity to the same two top-ranking type strains (*M. peregrinum* DSM 43271^T^ and *M. arcueilense* DSM 46715^T^) and 99.7% to the latter two reference strains. When compared with other *Mycobacterium* type strains, the sequence similarity values ranged from 98.5% to 99.1% for strain 19-857^T^ and from 99.0% to 99.5% for strain 20-525 (Table S2). Of note, the 16S rRNA gene sequence similarities between the two isolates and their closest relatives were all above the commonly accepted threshold of 98.65% for bacterial species demarcation [16], indicating that these sequences alone were insufficient for precise species delineation and that whole-genome approaches were required for further resolution.

**Figure 1:**
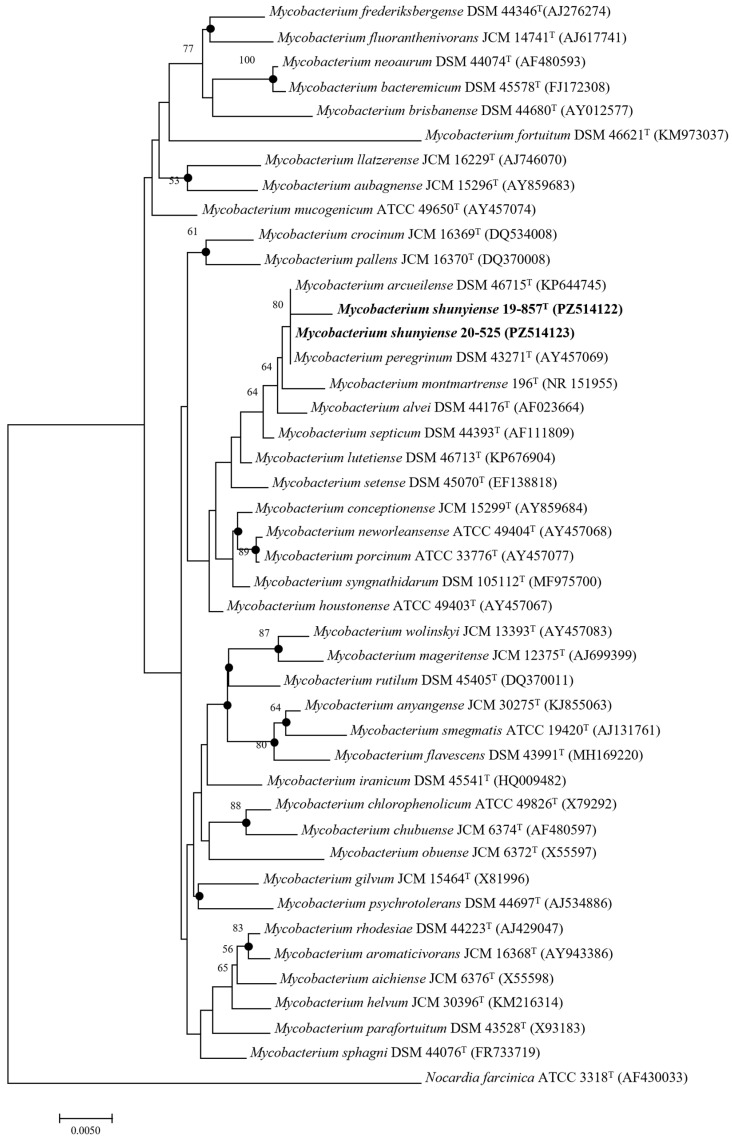
Neighbor-joining tree based on 16S rRNA gene sequences showing the relationships of strains 19-857^T^ and 20-525 with other representatives of the genus *Mycobacterium*. Filled circles indicate that the corresponding nodes were also recovered in phylogenetic trees generated using maximum likelihood and maximum parsimony methods. Bootstrap values above 50% are shown as percentages of 1000 replicates. *Nocardia farcinica* ATCC 3318^T^ (GenBank accession AF430033) was used as the outgroup. Scale bar indicates 0.005 nt substitutions per alignment site.

While 16S rRNA gene-based phylogeny is not suitable for species demarcation, it can reliably reveal the phylogenetic placement of isolates at the genus level. To this end, phylogenetic analysis based on 16S rRNA gene sequences placed both isolates within the genus *Mycobacterium*, clustering with nontuberculous mycobacteria. In the resulting phylogenetic tree, strains 19-857^T^, 20-525, *M. arcueilense* DSM 46715^T^, and *M. peregrinum* DSM 43271^T^ formed a well-supported clade (bootstrap value of 80%; Figure 1), reflecting their close evolutionary relatedness. This branching pattern was further corroborated by maximum likelihood and maximum parsimony analyses. A genome-based phylogeny reconstructed using the supermatrix approach recapitulated this topology (Figure S3), with the same four strains again clustering into a well-supported subclade, consistent with the 16S rRNA gene tree. Both the 16S rRNA gene-based tree (Figure 1) and the genome-based phylogenomic tree (Figure S3) recovered the same overall topology, with strains 19-857^T^ and 20-525 consistently clustering together within a subclade containing *M. peregrinum* DSM 43271^T^ and *M. arcueilense* DSM 46715^T^, confirming the robust phylogenetic placement of the two novel strains.

To further evaluate the genomic basis for species delineation, the quality and features of the draft genomes were first assessed. CheckM analysis estimated the genome completeness of strains 19-857^T^ and 20-525 at 99.8% and 100.0%, with contamination levels of 0.9% and 1.1%, respectively. The draft genome of strain 19-857^T^ comprised 48 contigs with a total length of 6,560,966 bp, an N50 of 386,284 bp, and a G+C content of 66.3%, whereas strain 20-525 consisted of 53 contigs spanning 7,283,373 bp, with an N50 of 386,284 bp and a G+C content of 66.0%. Annotation predicted 6361 coding sequences (CDSs) and 69 RNA genes (4 rRNAs, 3 ncRNAs, and 62 tRNAs) for strain 19-857^T^ and 7065 CDSs with 61 RNA genes (4 rRNAs, 3 ncRNAs, and 54 tRNAs) for strain 20-525 (Table S4). Subsequently, genomic relatedness between the two isolates and closely related *Mycobacterium* type strains was assessed via ANI and dDDH calculations (Table S5), yielding values of 85.8–94.1% and 29.1–54.3%, respectively—all below the established species delineation cutoffs (95–96% for ANI and 70% for dDDH) [17,18]. In contrast, the ANI and dDDH values between the two isolates themselves were 98.8% and 90.1%, respectively. Collectively, these genomic data confirmed that strains 19-857^T^ and 20-525 represented novel, previously undescribed species within the genus *Mycobacterium*.

To explore the genetic underpinnings of the observed resistance phenotypes in strains 19-857^T^ and 20-525, the genomes of both isolates were screened against the CARD database using both strict and loose algorithms. The strict algorithm identified three shared resistance determinants: *tap* (encoding an MFS efflux pump), *arr-1* (ADP-ribosyltransferase), and *aac(2′)-Ib* (aminoglycoside 2′-N-acetyltransferase). The loose algorithm further detected *tet(V)* (MFS efflux pump), *aph(3″)-Ic* (aminoglycoside phosphotransferase), *rpoB2* (rifamycin-resistant RNA polymerase beta-subunit), and *blaF* (family *β*-lactamase) in both isolates. To provide a comparative context for these findings, the genomes of the two reference strains, *M. arcueilense* DSM 46715^T^ and *M. peregrinum* DSM 43271^T^, were screened against the CARD database using the same criteria. Under the strict algorithm, *M. arcueilense* DSM 46715^T^ harbored *tap* and *aac(2′)-Ib*, while *M. peregrinum* DSM 43271^T^ harbored *tap*, *arr-1*, and a*ac(2′)-Ib*—a profile identical to that of *M. shunyiense*. Under the loose algorithm, both reference strains additionally harbored *tet(V)*, *aph(3″)-Ic*, *rpoB2*, and *blaF*, mirroring the accessory resistome of *M. shunyiense*. Thus, the resistance gene profiles of the reference strains were largely consistent with those of *M. shunyiense*, with the exception that *M. arcueilense* DSM 46715^T^ lacked arr-1 under the strict algorithm. The presence of *blaF* correlated with resistance to amoxicillin/clavulanate, while *tap* and *tet(V)* corresponded to resistance to minocycline and doxycycline. In addition, *arr-1* together with *rpoB2* provided a genetic basis for rifampicin resistance. Although *aac(2′)-Ib* and *aph(3″)-Ic* were indicative of aminoglycoside resistance potential, both strains remained susceptible to amikacin and tobramycin, indicating that these genes were not functionally expressed under the conditions tested (Table S2).

**Figure 2:**
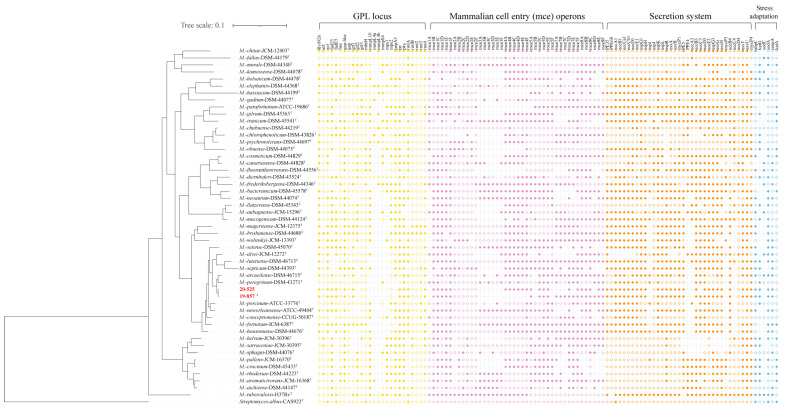
Predicted genetic determinants of virulence in strains 19-857^T^, 20-525, and phylogenetically related *Mycobacterium* strains.

Virulence-associated gene prediction revealed that strain 19-857^T^ harbored 258 such genes, while strain 20-525 contained 284, with the count ranging from 36 to 285 among phylogenetically related *Mycobacterium* strains (Figure 2). As integral components of the mycomembrane architecture, glycopeptidolipids (GPLs) are implicated in multiple pathogenic phenotypes, including sliding motility, biofilm development, host cell adherence, and intramacrophage persistence [19]. The GPL biosynthesis gene repertoire, encompassing *Rv0926*, *ecf*, *fad23*, *fadE5*, *gap-like*, *gtf1*, *gtf2*, *mmpL10*, *mmpL4a*, *mmpL4b*, *mmpS4*, *mps1*, *papA3*, *pe*, *pks*, *rmlA*, and *rmt4*, was present in both test strains. In addition, *mmpL11*, which is known to encode a transmembrane heme transporter, was also identified in both genomes of strains 19-857^T^ and 20-525, pointing to a potential alternative route for heme acquisition. Furthermore, both strains possessed a complete gene cluster for mycobactin synthesis, harboring the core enzymatic genes *mbtB*, *mbtD*, and *mbtE*. Given that mycobactin functions as a principal siderophore in mycobacteria for sequestering non-heme iron, the completeness of this biosynthetic pathway suggested that these organisms were equipped for siderophore-dependent iron scavenging.

The mammalian cell entry (*mce*) genes, which encode ABC transporters localized in the extracellular membrane for nutrient uptake, are critical for mycobacterial invasion of host cells. Genomic screening revealed that both strains 19-857^T^ and 20-525 commonly harbored six *mce* operons: *mce1*, *mce3*, *mce4*, *mce5*, *mce6*, and *mce7*. Among the well-characterized virulence factors of mycobacteria, the ESX secretion systems—also referred to as type VII secretion systems (T7SS)—were notably present in both isolates, a feature commonly observed among NTM. The *esx* genes detected in both isolates fell predominantly into three categories: ESX-1, ESX-3, and ESX-4.

CRISPR (clustered regularly interspaced short palindromic repeat) analysis identified four arrays in the genome of strain 20-525, suggesting that this isolate might have been exposed to multiple phage infections during its evolutionary history. In contrast, no CRISPR sequences were detected in strain 19-857^T^. Overall, 35 insertion sequences (ISs) were predicted from the two genomes combined, distributed among ten families: IS*3* (12 copies), IS*110* (8), IS*200*/IS*605* (4), IS*21* (3), IS*30* (2), IS*5* (2), IS*1182* (1), IS*481* (1), IS*630* (1), and IS*L3* (1) (Table S6). Additional transposase-related sequences were also identified in both genomes, with marked differences in their distribution patterns. Specifically, IS*C1316* elements, which belong to the IS200/IS605 family and were originally identified in Sulfolobus solfataricus, were more abundant, with three copies detected in strain 19-857^T^ and four in strain 20-525 (E-values: 7.0 × 10^−71^ to 4.0 × 10^−16^). In addition, two sequences matching the mariner transposon (a member of the Tc1/mariner superfamily of cut-and-paste DNA transposons that transpose via a TA-targeting mechanism and are among the most broadly distributed DNA transposons in nature [20]) were identified exclusively in strain 20-525, with E-values ranging from 3.0 × 10^−22^ to 2.0 × 10^−12^ (Table S7).

## 3. Discussion

In recent years, the number of recognized nontuberculous mycobacteria (NTM) species has risen substantially. Taxonomic methodologies have undergone continuous refinement—from morphological and cultural characterization, through numerical taxonomy and chemotaxonomy, to molecular and polyphasic approaches, and ultimately to genome-based taxonomy—which has markedly accelerated the rate at which novel species are being described. Concurrently, the widespread adoption of whole-genome sequencing has led to substantial revisions of phylogenetic relationships within the NTM group, and our understanding of these organisms has advanced in parallel. The majority of NTM species are saprophytes and pose little threat to human or animal health, with only a minority capable of triggering opportunistic infections under conducive conditions. Among the clinically significant members, clinically relevant NTM—such as the *M. avium* complex (MAC), the *M. abscessus* complex (MABC), and *M. kansasii*—are responsible for a rising global burden of pulmonary disease, a syndrome that accounts for the majority of NTM-associated clinical cases. Notably, the worldwide incidence and prevalence of pulmonary disease caused by multidrug-resistant NTM have also been on the rise [21,22]. By contrast, less virulent species, including *M. gordonae*, *M. terrae*, and the *M. fortuitum* complex, are typically considered as contaminants rather than true etiological agents of NTM pulmonary disease [22,23]. Consequently, precise identification of NTM isolates from clinical specimens is of critical importance, as the clinical significance of different NTM species varies considerably, depending on the proportion of patients with positive NTM cultures who ultimately fulfill the diagnostic criteria for NTM pulmonary disease [23].

Strains 19-857^T^ and 20-525 were recovered from sputum specimens of two male patients undergoing routine follow-up as part of initial treatment for suspected pulmonary tuberculosis in Beijing, China. The former was obtained from a 51-year-old patient in Shunyi District, who presented with chest pain and night sweats prior to treatment, along with radiographic evidence of patchy opacities of heterogeneous density in the right upper lobe. The latter originated from a 60-year-old patient in Pinggu District, who exhibited a productive cough and imaging findings of bilateral silicosis with cavitation in the right upper lobe, but without miliary nodules.

It should be noted that the present study does not establish a definitive causal relationship between *M. shunyiense* and the observed pulmonary lesions. NTM isolated from sputum may represent colonization, transient infection, or contamination, particularly in patients with underlying structural lung diseases. The patients from whom the strains were isolated had only single positive cultures at the time of strain collection and thus did not fully meet the ATS/IDSA diagnostic criteria for NTM pulmonary disease [24]. Treatment regimens and clinical outcomes were not systematically documented as this was a strain characterization study rather than a clinical cohort. Future clinical studies with longitudinal follow-up will be required to establish the pathogenic significance of *M. shunyiense*.

The nearly complete 16S rRNA gene sequences of both isolates were subsequently obtained via PCR amplification and sequencing. The 16S rRNA gene sequence-based identification revealed that strain 19-857^T^ shared 99.6% similarity with both *M. peregrinum* DSM 43271^T^ and *M. arcueilense* DSM 46715^T^, whereas strain 20-525 exhibited 100.0% similarity to both type strains. Given the high degree of sequence conservation in the 16S rRNA gene among closely related mycobacteria, these similarities alone were insufficient for accurate species delineation. Whole-genome comparisons based on ANI and dDDH were therefore undertaken to achieve higher taxonomic resolution. As expected, the ANI and dDDH values between the two isolates and their closest phylogenetic relatives ranged from 85.8% to 94.1% and from 29.1% to 54.3%, respectively—well below the widely accepted thresholds for bacterial species demarcation (95–96% for ANI and 70% for dDDH) [17,18]. In striking contrast, the genomic relatedness between the two isolates themselves reached 98.8% (ANI) and 90.1% (dDDH), substantially exceeding these boundaries. This marked discrepancy between high 16S rRNA gene similarity and low genome-based relatedness highlighted the importance of whole-genome approaches for accurate species delineation within the genus *Mycobacterium* [17]. Collectively, the genomic evidence strongly indicated that strains 19-857^T^ and 20-525 represent a single novel species within the genus *Mycobacterium*, distinct from all previously described taxa.

The fatty acid profiles of strains 19-857^T^ and 20-525 (Table S1) are broadly consistent with those of other rapidly growing mycobacteria, with C_18:1_*ω9c*, summed feature 3 (C_16:1_*ω7c*/C_16:1_*ω6c*), and C_14:0_ as the predominant components (each > 10%). Of particular interest is the presence of *anteiso*-C_17:0_ exclusively in strain 19-857^T^ (1.5%), which was not detected in strain 20-525 or the two reference strains. Branched-chain fatty acids such as *anteiso*-C_17:0_ are known to modulate membrane fluidity and permeability, potentially influencing bacterial adaptation to environmental stress, including antibiotic penetration and resistance. The differential fatty acid composition between the two isolates may contribute to their distinct phenotypic traits, such as the broader pH (6.0–9.0) and NaCl tolerance (0–7%) ranges observed in strain 20-525 (Table 1). Furthermore, variations in mycolic acid and fatty acid profiles have been implicated in host immune recognition and virulence in pathogenic mycobacteria [25]. Future studies correlating lipidomic profiles with stress tolerance and host–pathogen interactions will be valuable to elucidate the functional significance of these differences.

To further substantiate this conclusion, phenotypic and chemotaxonomic characterization of the two isolates was carried out in parallel. Phenotypic analysis revealed several distinctive traits that set strains 19-857^T^ and 20-525 apart from their closest phylogenetic neighbors (Table 1). Notably, strain 19-857^T^ possessed the additional ability to hydrolyze starch and cellulose. The chemotaxonomic profiles of both isolates were consistent with their placement in the genus *Mycobacterium*, lending further support to the findings derived from phylogenetic analyses. Moreover, both 16S rRNA gene-based and genome-derived protein sequence-based phylogenies consistently placed the two isolates together with *M. peregrinum* DSM 43271^T^ and *M. arcueilense* DSM 46715^T^ within a stable subclade. Taken together, the integrated phenotypic, chemotaxonomic, and phylogenomic data indicated that strains 19-857^T^ and 20-525 constitute a novel species within the genus *Mycobacterium*.

Although both isolates were obtained from patients with compatible clinical and radiographic findings, the present study does not establish a definitive causal relationship between *M. shunyiense* and the observed pulmonary lesions. NTM isolated from sputum may represent colonization, transient infection, or laboratory contamination, particularly in patients with underlying structural lung diseases. To our knowledge, this is the first report of *M. shunyiense*, and its formal pathogenic potential remains to be confirmed. Future studies employing experimental infection models (e.g., murine models) and clinical follow-up of patients with multiple positive cultures meeting the ATS/IDSA diagnostic criteria for NTM pulmonary disease will be required to establish its role as a genuine pathogen. Additionally, comparative genomic analysis with other clinically relevant NTM species may help identify specific virulence determinants that could serve as molecular markers for pathogenicity.

The identification of antimicrobial resistance determinants in mycobacterial genomes has become increasingly relevant for assessing the clinical significance of emerging NTM species [26,27]. In the current study, the resistance genes predicted by both strict and loose algorithms (as detailed in Section 2) encompassed determinants associated with multiple antibiotic classes, including rifampicin, tetracyclines, aminoglycosides, and *β*-lactams. Among the identified resistance genes, those conferring rifampicin resistance are particularly well characterized. The *arr-1* gene encodes a rifampicin ADP-ribosyltransferase that inactivates the antibiotic through ADP-ribosylation, as previously described in members of the genus *Mycobacterium* [26,27]. In parallel, the *rpoB2* variant, which contains multiple amino acid substitutions (≥8) within the rifampicin-binding site of the RNA polymerase *β*-subunit, renders the bacteria refractory to rifampicin by reducing drug-target affinity [27]. The coexistence of both *arr-1* and *rpoB2* in these isolates likely explains the strong rifampicin-resistant phenotype observed. With respect to tetracycline resistance, the tap and *tet(V)* genes both encode MFS-type efflux pumps that actively expel tetracycline from the cell in a proton-motive force-dependent manner, a mechanism previously reported in *M. smegmatis* and *M. fortuitum* [27]. The presence of these two efflux pumps is consistent with the resistance to minocycline and doxycycline observed phenotypically. For aminoglycosides, *aac(2′)-Ib* encodes an acetyltransferase that modifies the 2′-amino group of aminoglycoside substrates in a CoA-dependent manner, while *aph(3″)-Ic* encodes a phosphotransferase that phosphorylates aminoglycoside molecules, both leading to reduced ribosomal binding and subsequent antibiotic inactivation [26,28]. However, despite the presence of these genes, both isolates remained susceptible to amikacin and tobramycin in phenotypic tests, implying that these aminoglycoside resistance determinants are either not functionally expressed under the conditions tested or are insufficient to confer resistance independently. Finally, the detection of *blaF*, a *β*-lactamase originally characterized in *M. fortuitum* that hydrolyzes *β*-lactam antibiotics [25], provides a plausible genetic explanation for the observed resistance to amoxicillin/clavulanate. Overall, the resistome analysis revealed a complex and gene-specific correlation with the phenotypic susceptibility profiles. While the rifampicin-, tetracycline-, and *β*-lactam-associated resistance genes showed good concordance with the observed drug resistance, the presence of aminoglycoside resistance genes without corresponding phenotypic resistance highlights the frequent genotype–phenotype discordance in *Mycobacterium*, which might be attributed to gene silencing, insufficient expression levels, or redundancy in resistance pathways.

The resistance gene profiles of *M. shunyiense* are largely consistent with those of its closest phylogenetic relatives, *M. peregrinum* and *M. arcueilense*, suggesting a conserved resistome within this clade. Under the strict algorithm, *M. shunyiense* and *M. peregrinum* share an identical repertoire of resistance determinants (*tap*, *arr-1*, and *aac(2′)-Ib*), while *M. arcueilense* lacks *arr-1*. The presence of *arr-1*, encoding a rifampicin ADP-ribosyltransferase, together with *rpoB2*, likely explains the rifampicin resistance phenotype observed in *M. shunyiense* and suggests that *M. peregrinum* may possess a similar resistance potential given its identical *arr-1/rpoB2* gene repertoire, whereas *M. arcueilense* may rely solely on *rpoB2*-mediated resistance if phenotypically resistant. Under the loose algorithm, all three species share the same set of resistance genes, including *tet(V)*, *aph(3″)-Ic*, and *blaF*, indicating a conserved accessory resistome within this phylogenetic clade. This comparison highlights the value of genome-based resistome analysis for predicting antimicrobial susceptibility patterns in emerging NTM species and provides a genomic context for interpreting the resistance potential of *M. shunyiense* relative to its close relatives.

Pathogenic mycobacteria produce a broad spectrum of lipids that are critically involved in host–pathogen interactions and virulence [29,30,31]. Glycopeptidolipids (GPLs), by contrast, are found exclusively in only a limited subset of mycobacterial species, yet they fulfill multiple functions in environmental survival and pathogenesis [29,30]. Previous studies have demonstrated that GPLs are associated with sliding motility and biofilm formation in *M. smegmatis* and *M. avium*, traits that are thought to facilitate colonization of both abiotic surfaces and host tissues [31,32]. In *M. abscessus*, spontaneous transitions between smooth and rough colony morphotypes have been correlated with GPL expression levels: smooth variants produced abundant GPLs and exhibited sliding motility and biofilm formation, whereas rough variants expressed minimal GPLs and lost these surface-associated phenotypes [33]. These observations suggested that GPLs might serve as a “colonization factor” that facilitates environmental persistence while simultaneously masking or interfering with the expression of core virulence determinants, such as those involved in cording. In line with this concept, the GPL-related gene repertoire identified in strains 19-857^T^ and 20-525—including *Rv0926*, *mmpL4a*, *mmpL4b*, *mps1*, *pks*, and *rmlA*—suggested that these isolates harbor the genetic machinery for GPL biosynthesis. However, the mere presence of these genes does not necessarily reflect the level of GPL expression, given that phenotypic switching can occur through reversible regulatory mechanisms independent of gene deletion [33]. Therefore, further investigation is needed to determine the actual GPL production and its potential impact on the pathogenic behavior of strains 19-857^T^ and 20-525. To complement the genomic predictions, future work should focus on phenotype-based validation experiments. Specifically, the actual GPL production could be assessed through lipid extraction and TLC or MALDI-TOF mass spectrometry analysis under various culture conditions (e.g., different temperatures, nutrient availability, and biofilm-inducing media). Functional assays examining sliding motility on semisolid agar, biofilm formation on abiotic surfaces, and adherence to host epithelial cells in vitro would clarify the biological consequences of GPL expression. Additionally, comparative analyses between strains 19-857^T^ and 20-525, which show differential phenotypic traits (e.g., salt tolerance and hydrolytic activities, as shown in Table 1), may help correlate GPL expression levels with specific phenotypic outcomes. These experiments will be critical in determining whether GPLs serve as a ‘colonization factor’ in *M. shunyiense*, as previously demonstrated in *M. smegmatis* and *M. abscessus*.

In addition to surface-associated lipids such as GPLs, protein secretion systems and nutrient uptake machineries also constitute key determinants of mycobacterial pathogenicity. Among these, the ESX secretion systems (type VII secretion systems, T7SS) are particularly notable for their involvement in substrate transport and host interactions. Genomic screening of strains 19-857^T^ and 20-525 revealed that the *esx* genes detected in both isolates fell predominantly into three categories: ESX-1, ESX-3, and ESX-4. The ESX-1 system is known to export immunogenic protein effectors that contribute to virulence [34]. ESX-3 has been associated with siderophore-dependent iron acquisition, whereas ESX-4 is regarded as the ancestral ESX lineage, from which other subtypes are thought to have arisen through gene duplication and subsequent functional divergence, and it has also been implicated in phagosomal disruption within host macrophages [35]. In addition to these secretion systems, both strains harbored a complete set of six *mce* operons (*mce1*, *mce3*, *mce4*, *mce5*, *mce6*, and *mce7*), which encode ABC transporters located in the extracellular membrane for nutrient uptake and are critical for mycobacterial invasion of host cells. The presence of multiple *mce* operons in these isolates implies a broad capacity for nutrient acquisition and host cell interaction, consistent with the pathogenic potential observed in other NTM species.

Beyond the canonical virulence determinants discussed above, the genomic architecture of these two isolates also revealed notable features pertaining to genome plasticity and evolutionary adaptation. CRISPR (clustered regularly interspaced short palindromic repeat) analysis identified four arrays in the genome of strain 20-525, suggesting that this isolate may have encountered multiple phage infections during its evolutionary history, whereas the complete absence of CRISPR sequences in strain 19-857^T^ pointed to a differential phage–host interaction history between the two strains. This disparity is of particular interest, as CRISPR-Cas systems are known to provide adaptive immunity against phage invasion [36,37] and also play roles in modulating horizontal gene transfer and shaping accessory genomes in prokaryotes [38]. The presence of CRISPR arrays in one isolate but not the other may therefore reflect different ecological niches or disparate exposure histories experienced by these two strains prior to isolation.

A total of 35 insertion sequences (ISs) were predicted from the two genomes combined, distributed across ten families, with the most abundant being IS*3* (12 copies), IS*110* (8), and IS*200*/IS*605* (4). IS elements are known to drive genomic rearrangements, gene inactivation, and the acquisition of novel functions, thereby contributing to mycobacterial genome plasticity and adaptive evolution [39]. In *Mycobacterium tuberculosis*, insertion sequences such as IS*6110* have been shown to generate structural diversity within CRISPR loci through recombination events, leading to deletions and duplications [40,41]. The high IS load observed in both isolates is consistent with the notion that the *Mycobacterium* genome contains a substantial mobile genetic element repertoire, which may facilitate rapid adaptation to fluctuating environmental conditions. Notably, the distribution of IS elements and transposase-related sequences differed between the two strains. For instance, mariner transposon sequences were detected exclusively in strain 20-525. These differences in mobile genetic element distribution suggested that the two isolates may have undergone distinct genomic trajectories after diverging from a common ancestor, potentially contributing to phenotypic differences observed in their GPL expression profiles or metabolic capabilities. However, whether these differences have direct implications for virulence or host adaptation remains to be determined through further experimental investigation.

The two *M. shunyiense* strains, despite sharing high genomic relatedness (98.8% ANI), exhibit notable differences in several physiological and genomic features that may reflect distinct ecological adaptations. Strain 20-525 displayed a broader pH tolerance range (up to pH 9.0) and higher NaCl tolerance (up to 7.0%) compared to strain 19-857^T^ (pH 6.0–8.0, NaCl up to 6.0%), suggesting enhanced capacity to withstand alkaline and osmotic stress. These differences may be attributed to strain-specific genetic determinants, including the presence of CRISPR arrays exclusively in strain 20-525 (four arrays identified), which implies a history of phage exposure and potentially enhanced adaptive immunity. Additionally, the differential fatty acid profiles—notably the exclusive presence of *anteiso*-C_17:0_ in strain 19-857^T^—may contribute to variations in membrane fluidity and permeability, affecting stress tolerance. The presence of distinct mobile genetic element distributions further suggests that the two strains have undergone divergent evolutionary trajectories, potentially driven by different environmental niches prior to isolation. These phenotypic and genotypic variations highlight the intraspecies diversity within *M. shunyiense* and may reflect adaptive responses to different microenvironments encountered during their ecological history. Future studies employing comparative transcriptomics or phenotypic microarrays would be valuable to further elucidate the functional significance of these differences.

## 4. Materials and Methods

### 4.1. Strain Acquisition

Strains 19-857^T^ and 20-525 were isolated from sputum specimens of two patients with suspected pulmonary tuberculosis in Beijing, China. Specifically, strain 19-857^T^ was obtained from a patient with pulmonary tuberculosis in Shunyi District, Beijing. Prior to treatment, the patient presented with chest pain and night sweats; chest X-ray revealed patchy shadows of heterogeneous density in the upper lobe of the right lung. Strain 20-525 was isolated from another patient with pulmonary tuberculosis in Pinggu District, Beijing, who had experienced productive cough for more than two weeks before treatment. Chest X-ray showed bilateral silicotic changes and a cavity in the upper lobe of the right lung, but no miliary pattern. Ziehl–Neelsen staining of the sputum smears from both patients yielded negative results for acid-fast bacilli.

For isolation, 1–2 mL of sputum was mixed with an equal volume of 4% NaOH (1–2 times the sample volume) and vortexed for 30 s, followed by a 15 min incubation at room temperature. Subsequently, 0.1–0.15 mL of the treated specimen was inoculated onto Löwenstein–Jensen medium and incubated at 37 °C for three weeks. Visible colonies were then picked and streaked onto tryptic soy agar (TSA; Difco, Becton, Dickinson and Company, Franklin Lakes, NJ, USA), and the cultures were further incubated at 37 °C to obtain isolated colonies.

Purified isolates were maintained in glycerol suspensions (20%, *v*/*v*) at −80 °C.

The reference strains *M. peregrinum* DSM 43271^T^ and *M. arcueilense* DSM 46715^T^ were acquired from the German Collection of Microorganisms and Cell Cultures (DSMZ).

### 4.2. Phenotypic, Physiological and Biochemical Characteristics

For morphological observation, strains 19-857^T^ and 20-525 were cultivated on TSA at 37 °C for five days. The harvested cells were then dispersed by mild ultrasonication, dropped onto silicon wafers, and thoroughly air-dried before being sputter-coated with gold and examined under a field-emission scanning electron microscope (FESEM; HITACHI SU8020, Hitachi High-Technologies Corporation, Tokyo, Japan). Biochemical phenotypes of the two isolates, together with the reference strains *M. peregrinum* DSM 43271^T^ and *M. arcueilense* DSM 46715^T^, were assessed in independent duplicate assays. Growth and pigmentation were evaluated on various media, including glucose–yeast extract–malt extract (GYM; DSMZ medium 65, https://www.dsmz.de/microorganisms/medium/pdf/DSMZ_Medium65.pdf accessed on 1 June 2026), nutrient agar (NA; Difco, Becton, Dickinson and Company, Franklin Lakes, USA), Reasoner’s 2A (R2A) agar (Difco, Becton, Dickinson and Company, Franklin Lakes, USA), tryptic soy agar (TSA; Oxoid, Thermo Fisher Scientific, Basingstoke, UK), Löwenstein-Jensen (L-J) medium, and Middlebrook 7H10 agar (MB7H10) [42] after incubation at 37 °C for five days. Gram staining was performed according to standard procedures. Catalase activity was determined by observing bubble production after the addition of 3% (*v*/*v*) hydrogen peroxide, and oxidase activity was measured using the API oxidase reagent (bioMérieux, Marcy-l’Étoile, Frence) according to the manufacturer’s instructions [43]. Nitrate reduction, hydrogen sulfide production, and hydrolysis of starch, gelatin, and cellulose were assessed using a biochemical identification kit (Huankai Microbial) following the manufacturer’s directions. Growth temperature range (4, 10, 15, 20, 25, 28, 30, 35, 37, 40, 45, and 50 °C), NaCl tolerance (0–12%, *w*/*v*, at 1% intervals), and pH range (pH 4–10 at 1 pH unit intervals) were examined using TSA as the basal medium at 37 °C for five days, with pH adjustment carried out using the buffer system described previously [44]; NaCl tolerance was specifically determined at 28 °C in TSA medium (pH 7.0, without Na^+^ and Cl^−^) supplemented with NaCl at concentrations ranging from 0 to 10.0% (*w*/*v*) in 1.0% increments. All growth condition tests were performed in triplicate. The Biolog GEN III MicroPlate system was used to analyze sensitivity to various chemical reagents, including antibiotics, as well as the utilization of carbon and nitrogen sources. Enzymatic activities were characterized using the API ZYM system according to the manufacturer’s instructions, and acid production from fermentation was further evaluated using the API 50CH system. Results from API strips and Biolog plates were recorded every 48 h during incubation at 37 °C until stabilization, with all assays conducted in duplicate under consistent conditions.

### 4.3. Drug Resistance Test

Drug susceptibility testing was carried out using commercial kits specifically designed for nontuberculous mycobacteria (BASO, Zhuhai, China), according to a standardized broth microdilution method. All procedures strictly adhered to the manufacturer’s instructions, and *M. peregrinum* DSM 43271^T^ served as the quality control strain. The panel of antimicrobial agents tested included amikacin, amoxicillin/clavulanate, cefoxitin, minocycline, doxycycline, ciprofloxacin, clarithromycin, rifampicin, imipenem, linezolid, moxifloxacin, tigecycline, meropenem, tobramycin, trimethoprim/sulfamethoxazole, and rifabutin. The BASO NTM susceptibility testing system has been employed in recent studies for the antimicrobial susceptibility testing of clinically relevant NTM species [45]. To prepare the inoculum, 2–5 mg of bacterial biomass was harvested from colonies grown on solid medium and transferred to a grinding tube containing glass beads and sterile buffer. Following thorough vortexing, the suspension was adjusted to a turbidity equivalent to a 0.5 McFarland standard using either a nephelometer or visual comparison with a 0.5 McFarland standard tube. A 60 μL aliquot of the adjusted suspension was then added to 12 mL of inoculation broth and mixed well by vortexing. Subsequently, 100 μL of this diluted cell suspension was dispensed into each well of the drug susceptibility plate. The plates were incubated at 28 °C for 3 to 5 days, after which bacterial growth was examined and interpreted following the manufacturer’s directions.

### 4.4. Chemotaxonomic Characterization

For chemical analyses, strains 19-857^T^ and 20-525, together with the reference strains *M. peregrinum* DSM 43271^T^ and *M. arcueilense* DSM 46715^T^, were grown as pure cultures on TSA at 28 °C for 5 days prior to extraction. Polar lipids were extracted from wet cell biomass using a chloroform/methanol system and subsequently separated by two-dimensional thin-layer chromatography on TLC silica-gel 60 F_254_ plates (10 × 10 cm, Merck KGaA, Darmstadt, Germany), following the protocol originally described by Minnikin et al. [44]. The first dimension was developed with a chloroform/methanol/water mixture (64:25:5, by volume), and the second dimension with a chloroform/acetic acid/methanol/water mixture (80:18:12:5, by volume). Various polar lipid components were visualized using specific staining reagents, including phosphomolybdic acid for total lipids, molybdenum blue for phosphates, α-naphthol/sulfuric acid for carbohydrates, and ninhydrin for amino groups. For menaquinone extraction and purification, reverse-phase HPLC was employed following the procedures described in a previous study [46]. Fatty acid composition was analyzed using the Sherlock Microbial Identification System (MIDI) with the standard ACTIN1 database (version 6.0), and all operations were performed in strict accordance with the manufacturer’s guidelines [47]. The peptide subunit composition of the peptidoglycan was determined using the method originally described by Schleifer and Kandler. To identify the diaminopimelic acid isomer present in the cell wall, whole-cell hydrolysates (obtained by treatment with 6 N HCl at 120 °C for 30 min) were separated via thin-layer chromatography on cellulose plates (10 × 20 cm, Merck) using a solvent system previously described in the literature [48].

### 4.5. Phylogenetic Analysis and Genomic Characteristics

Genomic DNA was extracted from each bacterial isolate following dispersal of a 400 µL cell suspension by ultrasonication, using a modified CTAB (cetyltrimethylammonium bromide) protocol. DNA concentration and quality were assessed with a Qubit 2.0 Fluorometer (Thermo Fisher Scientific, Basingstoke, UK). The 16S rRNA gene was amplified via PCR with primers 27F and 1523R.

For preliminary taxonomic assignment, the resulting 16S rRNA gene sequences were compared against reference sequences available in the EzBioCloud database (www.ezbiocloud.net/). Sequence alignment was carried out with Clustal W [49], and phylogenetic trees were reconstructed using MEGA 12 [50] via the neighbor-joining method, with maximum parsimony and maximum likelihood approaches additionally applied for cross-validation. Nodal support was evaluated by bootstrap resampling (1000 replicates) [51], and genetic distances were computed employing the Kimura two-parameter model [52].

Whole-genome shotgun sequencing of strains 19-857^T^ and 20-525 was performed on the Illumina HiSeq 4000 platform, yielding 2 × 150 bp paired-end reads. De novo assembly was accomplished using SOAPdenovo v2.04 [53], followed by sequence refinement with CISA v1.3 [54], gap filling via GapCloser v1.12 [53], and elimination of contigs shorter than 500 bp. Raw FASTQ sequencing reads were quality-filtered using fastp (v0.20.0) to remove adapter sequences, duplicate reads, and low-quality bases (defined as a Phred quality score < 20 in more than 30% of the bases). The completeness and contamination levels of the assembled genomes were evaluated with the CheckM pipeline [55]. Taxonomic assignment at the species level was achieved through overall genome relatedness indices (OGRIs) and phylogenomic reconstruction. Average nucleotide identity (ANI) values between the two isolates and other Mycobacterium species were determined using FastANI v2.2.23, while digital DNA–DNA hybridization (dDDH) estimates were obtained from the Genome-to-Genome Distance Calculator 3.0 applying formula 2 [56]. In addition, a genome-based phylogenetic tree was reconstructed via the supermatrix approach, employing the bac120 gene set—a collection of 120 single-copy protein sequences widely distributed among bacteria. This analysis was performed with EasyCGTree 4 following the protocol described previously [57]. Evolutionary distances for this tree were computed using IQ-Tree 2 [58], for which the genome sequences of relevant strains were retrieved from the NCBI genome database.

Draft genome annotation was conducted with Prokka v1.13.3 under default parameters [59]. Screening for antimicrobial resistance genes was performed with RGI v6.0.3 against CARD (Comprehensive Antibiotic Resistance Database) (v3.2.4) [60], and virulence factors were identified via ABRicate v1.0.1 with the VFDB (Virulence Factors Database) [61] using default thresholds (≥80% identity and ≥80% coverage for ABRicate; both strict and loose criteria for RGI as per CARD guidelines). Mobile genetic elements were surveyed using a suite of tools including MOB-suite v3.1.9 [62], geNomad v1.7.1, IslandPath-DIMOB v1.0.0 [63], TransposonPSI v1.0, ISEScan v1.7.2.3 [64], and CRISPRCasFinder v4.2.19 [65].

## 5. Conclusions

In this study, two rapidly growing, non-pigmented mycobacterial strains, 19-857^T^ and 20-525, were isolated from sputum specimens of patients with suspected pulmonary tuberculosis in Beijing, China. Comprehensive polyphasic taxonomic characterization—including phenotypic, physiological, biochemical, chemotaxonomic, and phylogenomic analyses—clearly distinguished these isolates from their closest phylogenetic neighbors, *M. peregrinum* DSM 43271^T^ and *M. arcueilense* DSM 46715^T^. The ANI and dDDH values between the two isolates and all known Mycobacterium species were well below the established species delineation thresholds, while the high genomic relatedness between the two isolates themselves (98.8% ANI and 90.1% dDDH) confirmed their co-assignment to a single novel species. Based on these lines of evidence, we formally propose the classification of strains 19-857^T^ and 20-525 as *Mycobacterium shunyiense* sp. nov., with strain 19-857^T^ (=CGMCC 38906^T^) designated as the type strain. This discovery adds to the growing list of nontuberculous mycobacteria associated with respiratory infections, underscoring the clinical relevance of these organisms in individuals with predisposing lung conditions. The genomic analysis further revealed a rich repertoire of virulence-associated determinants, including GPL biosynthesis genes, complete mce and ESX secretion system operons, as well as multiple antimicrobial resistance genes, providing insights into the pathogenic potential and drug resistance mechanisms of this novel species. The presence of CRISPR arrays and a substantial load of insertion sequences in the genomes also highlighted the genomic plasticity and evolutionary adaptability of these isolates. Overall, the discovery of *M. shunyiense* expands the current understanding of NTM diversity in clinical settings and, more importantly, provides valuable reference data and diagnostic markers for the accurate identification of NTM species in clinical laboratories, which is essential for guiding appropriate therapeutic strategies and improving patient outcomes.

### Description of Mycobacterium Shunyiense sp. nov.

(shun.yi.en’se. N.L. neut. adj. *shunyiense*, pertaining to Shunyi, a district in Beijing, China, where the clinical specimens were collected from patients with suspected pulmonary tuberculosis.)

Cells were Gram-stain-positive, rod-shaped, and nonspore-forming. After 5 days of incubation at 28 °C, colonies appeared orange on L-J and TSA medium and cream-white on MB7H10, GYM and NA media, with only scant growth observed on R2A agar. Growths appear at 25–40 °C (optimum, 28 °C), pH 6–8 (optimum, pH 7) and with 0–6.0% NaCl (*w*/*v*). The strain was positive for catalase and negative for oxidase, gelatin hydrolysis, and H_2_S production. Acetic acid, D-fructose, D-fructose-6-PO_4_, D-gluconic acid, D-mannitol, D-saccharic acid, glucuronamide, L-malic acid, propionic acid, tween 40, *α*-hydroxy-butyric acid, and *α*-keto-butyric acid can be used as carbon substrates. Enzymatic activity analysis revealed the presence of acid phosphatase, cystine arylamidase, esterase (C4), esterase lipase (C8), leucine arylamidase, lipase (C14) and *β*-glucosidase. The polar lipids include diphosphatidylglycerol (DPG), phosphatidylethanolamine (PE), phosphatidylinositol (PI) and phosphatidylinositol mannosides (PIMs). Whole-cell hydrolysates contain *meso*-diaminopimelic acid (*meso*-A2pm). Major fatty acids (>10%) are C_18:1_*ω9c* (26.5%), summed feature 3 (20.4%) and C_14:0_ (12.6%). The primary menaquinone was MK-9 (H_2_). The type strain 19-857^T^ (=CGMCC 38906^T^) was isolated from the sputum of a suspected pulmonary tuberculosis patient in Shunyi District, Beijing, China. The genome size of the type strain is 6.6 Mb with a G+C content of 66.3%.

## Figures and Tables

**Table 1:** Differential characteristics of strains 19-857^T^, 20-525, and the reference strains *M. peregrinum* DSM 43271^T^ and *M. arcueilense* DSM 46715^T^.

Characteristic	1	2	3	4
Colony color on TSA	Orange	Orange	Creamy white	Creamy white
NaCl range for growth	0–6%	0–7%	0–6%	0–8%
pH range for growth	6–8	6–9	6–8	6–10
Nitrate reduced to nitrite	+	−	+	−
Hydrolysis of				
starch	+	−	−	−
Cellulose	+	−	−	−
Carbon sources used for growth				
D-trehalose	−	+	−	+
*α*-D-glucose	−	w	−	w
D-mannose	−	w	−	−
D-fructose	+	+	w	+
D-mannitol	+	+	w	+
D-fructose-6-PO_4_	+	w	w	+
L-alanine	−	w	−	+
L-arginine	−	w	−	+
L-glutamic acid	w	+	−	+
L-serine	−	−	−	+
L-galactonic acid lactone	−	−	−	+
D-gluconic acid	+	w	+	+
Glucuronamide	+	w	+	+
D-saccharic acid	+	−	+	−
D-lactic acid methyl ester	−	w	−	+
L-lactic acid	−	−	−	+
tween 40	+	+	w	+
*γ*-amino-butryric acid	−	−	−	+
*α*-hydroxy-butyric acid	+	+	w	+
*α*-keto-butyric acid	+	+	−	+
Formic acid	w	w	−	+
API ZYM results				
Alkaline phosphatase	w	−	+	−
Valine arylamidase	+	+	w	+
Trypsin	+	−	+	+
*α*-chymotrypsin	+	−	+	w
Naphthol-AS-BI-phosphohydrolase	+	+	+	w
*α*-galactosidase	w	−	w	−
*α*-glucosidase	w	−	−	−
N-acetyl-*β*-glucosamimidase	w	−	−	−
Acid production from substrates (API 50CH)				
Glycerol	−	+	−	+
Potassium 2-ketogluconate	+	−	−	-
*arr-1* resistance gene (strict algorithm)	+	+	+	−
*anteiso*-C_17:0_ fatty acid	+	−	−	−

Strains: 1, 19-857^T^; 2, 20-525; 3. *M. peregrinum* DSM 43271^T^; 4, *M. arcueilense* DSM 46715^T^. +, positive; w, weakly positive; −, negative activities/growth.

## Data Availability

The GenBank accession numbers for the 16S rRNA gene sequences of strains 19-857^T^ and 20-525 are PZ514122 and PZ514123, respectively; accession numbers for the genome sequences of strains 19-857^T^ and 20-525 are JBZDPT000000000 and JBZDPU000000000, respectively.

## Supplementary Materials

The following supporting information can be downloaded at https://www.mdpi.com/article/10.3390/ijms27156890/s1.
