# Supplementary material for: Genomic Characterization and Taxonomic Classification of Mycobacterium shunyiense sp. nov., a Novel Nontuberculous Mycobacterial Species from a Human Respiratory Specimen
Source: Int J Mol Sci. 2026 Aug 1;27(15):6890. doi: 10.3390/ijms27156890 (PMC13466811; doi:10.3390/ijms27156890)
Supplement: Supplementary file 1 [file ijms-27-06890-s001.zip › ijms-4447275-supplementary.pdf]

Genomic characterization and taxonomic classification of *Mycobacterium shunyiense* sp. nov., a novel nontuberculous mycobacterial species from a human respiratory specimen

Yang Deng[1,2], Xin-Yu Yang[1,2], Nen-Han Wang[1], Bo Li[1], Hong-Hao Miao[1], Jie Zhang[1,2], Meng-Di Pang[1,2], Yi-Xuan Ren[1], Jun-Li Yi[1], Man Cai[3,*] and Jie Li[1,2,*]

1. Beijing Center for Disease Prevention and Control, Beijing, 100013, PR China.
2. School of Public Health, Capital Medical University, Beijing, 100069, PR China.
3. Institute of Microbiology, China General Microbiological Culture Collection Center, Chinese Academy of Sciences, Beijing, 100101, PR China.

Correspondence: Man Cai, caiman@im.ac.cn; Jie Li, lijie304304@163.com.

Figure S1. Scanning electron microscopy of strains 19-857^T (A) and 20-525 (B)

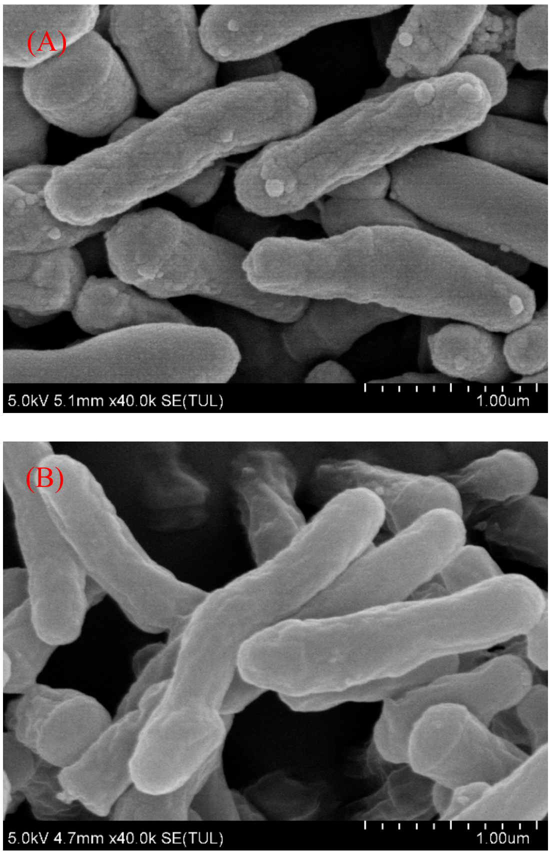

Figure S2. Two-dimensional thin-layer chromatography of polar lipids of strains 19-857^T and 20-525.

Detection is shown after spraying with (a) molybdatophosphoric acid reagent; (b) ninhydrin

stain reagent (c); molybdenum blue stain reagent; and (d) α-naphthol/sulfuric acid stain reagent.

DPG, diphosphatidylglycerol; PE, phosphatidylethanolamine; PI, phosphatidylinositol; GL, glycolipid; PIM, phosphatidylinositol mannosides.

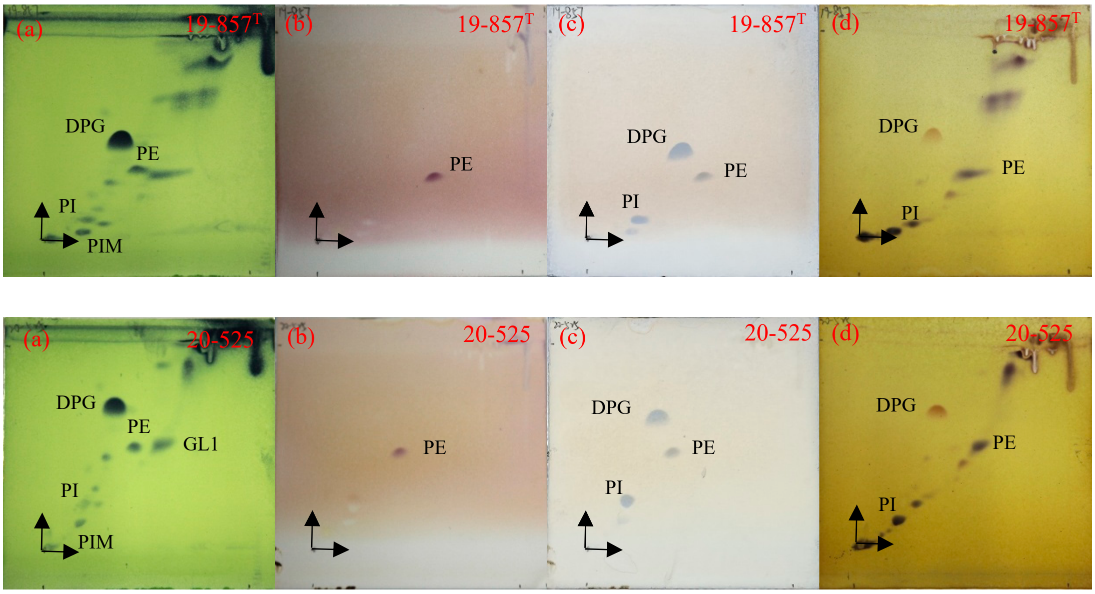

Figure S3. Maximum-Likelihood phylogenomic tree showing the phylogenetic positions of strains 19-525^T and 20-525 within the *Mycobacterium* genus based on conserved core genes.

Bootstrap values are shown at branch nodes as a percentage of 1,000 bootstrap replicates. RefSeq assembly accession numbers for *Mycobacterium* references are shown in brackets. *Nocardia farcinica* ATCC 3318^T (RefSeq assembly accession no. GCF 001612925.1) was used as the outgroup (not shown in the figure). Bar indicates 0.04 substitutions per nucleotide position.

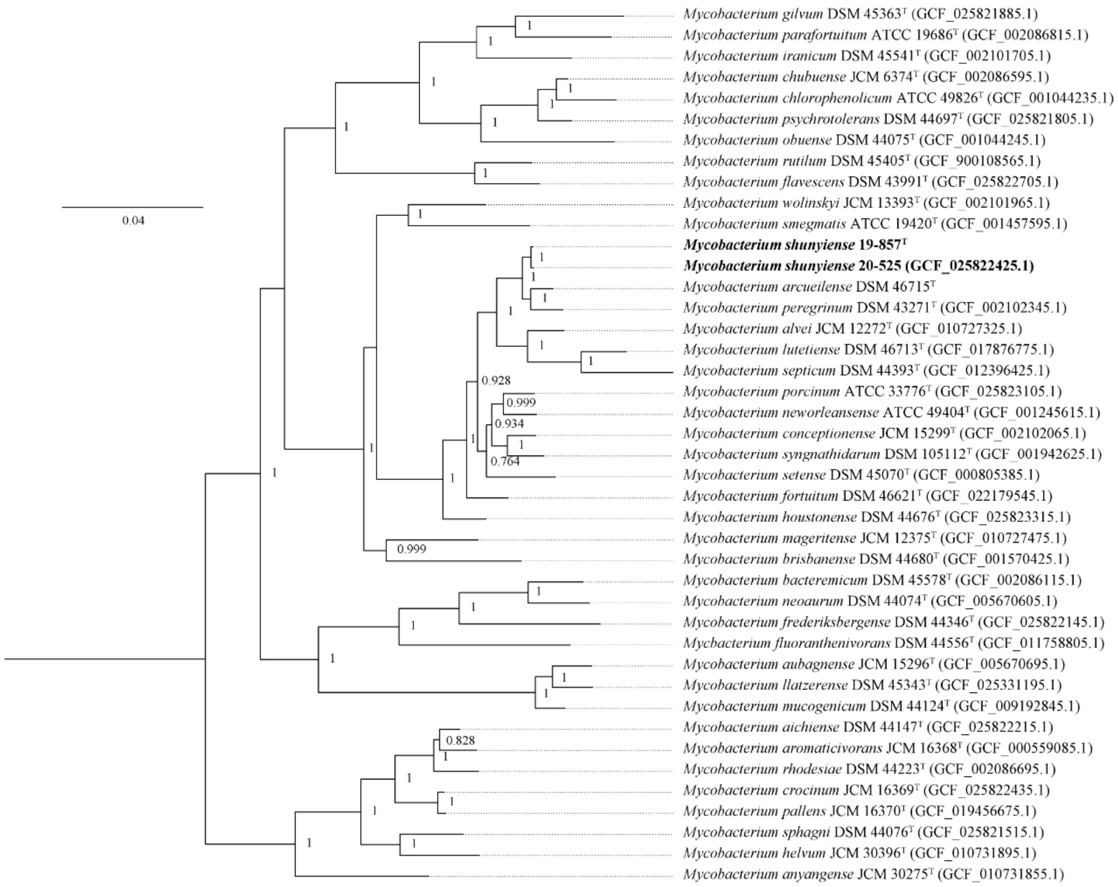

Table S1. Cellular fatty acid profiles of strains 19-857^{T} and 20-525, along with the reference strains of genus *Mycobacterium*.

Strains: 1, 19-857^{T}; 2, 20-525; 3. *M. peregrinum* DSM 43271^{T}; 4, *M. arcueilense* DSM 46715^{T}.

Fatty acid (%)	1	2	3	4
**Saturated**				
$C_{14:0}$	**12.6**	**12.1**	**13.0**	**11.4**
$C_{18:0}$	1.5	1.3	1.4	1.3
**Branched**				
anteiso-$C_{17:0}$	1.5	-	-	-
$C_{18:0}$ 10-methyl	2.2	2.1	3.8	3.0
**Unsaturated**				
$C_{17:0}$ 3-OH	2.0	1.6	1.2	-
$C_{16:1}\omega 9c$	-	1.7	1.6	2.1

$C_{16:1}\omega 11c$	1.8	-	-	-
$C_{17:1}\omega 8c$	1.4	1.3	1.0	-
$C_{18:1}\omega 9c$	**26.5**	**27.8**	**25.1**	**29.3**
**Summed features**				
3 ($C_{16:1}\omega 7c$/$C_{16:1}\omega 6c$)	**20.4**	**20.7**	**22.0**	**20.9**

Table S2. Antimicrobial susceptibility testing result of strains 19-857^T and 20-525.

Antibiotic	Susceptible (S) (μg/mL)	Intermediate (I) (μg/mL)	Resistant (R) (μg/mL)	MIC of 19-857^T (μg/mL)	Interpretation of 19-857^T	MIC of 20-525 (μg/mL)	Interpretation of 20-525
amikacin	≤16	32	≥64	≤2	S	≤2	S
amoxicillin/clavulanate	≤8/4	16/8	≥32/16	>64/32	R	>64/32	R
cefoxitin	≤16	32-64	≥128	16	S	16	S
minocycline	≤1	2-4	≥8	>8	R	8	R
doxycycline	≤1	2-4	≥8	>16	R	>16	R
ciprofloxacin	≤1	2	≥4	≤0.12	S	≤0.12	S
clarithromycin	≤2	4	≥8	≤1	S	≤1	S
rifampicin	≤1	-	≥2	>4	R	>4	R
imipenem	≤4	8-16	≥32	4	S	4	S
linezolid	≤8	16	≥32	8	S	4	S
moxifloxacin	≤1	2	≥4	≤0.25	S	≤0.25	S
tigecycline	≤4	-	>4	≤0.25	S	≤0.25	S
meropenem	≤4	8-16	≥32	4	S	4	S
tobramycin	≤2	4	≥8	4	S	4	S
trimethoprim/sulfamethoxazole	≤2/38	-	≥4/76	≤0.25/4.8	S	0.5/9.5	S
rifabutin	≤2	-	≥4	1	S	1	S

Table S3. 16S rRNA gene nucleotide sequence similarities (%) shared between genes of strains 19-857^T, 20-525, and phylogenetically-related *Mycobacterium* strains.

Species designations are as follows: 1, 19-857^T; 2, 20-525; 3, *M. arcueilense* DSM 46715^T; 4, *M. peregrinum* DSM 43271^T; 5, *M. montmartrense* 196^T; 6, *M. alvei* DSM 44176^T; 7, *M. septicum* DSM 44393^T; 8, *M. lutetiense* DSM 46713^T; 9, *M. setense* DSM 45070^T; 10, *M. conceptionense* JCM 15299^T; 11, *M. neworleansense* ATCC 49404^T; 12, *M. porcinum* ATCC 33776^T; 13, *M. syngnathidarum* DSM 105112^T;14, *M. houstonense* ATCC 49403^T.

species	1	2	3	4	5	6	7	8	9	10	11	12	13	14
1	100.0													
2	99.6	100.0												
3	99.6	100.0	100.0											
4	99.6	100.0	100.0	100.0										
5	99.1	99.5	99.5	99.5	100.0									
6	98.7	99.1	99.1	99.1	98.6	100.0								
7	99.3	99.7	99.7	99.7	99.2	99.4	100.0							
8	99.0	99.4	99.4	99.4	99.0	99.2	99.7	100.0						
9	98.7	99.1	99.1	99.1	98.6	99.1	99.4	99.7	100.0					
10	98.8	99.2	99.2	99.2	98.9	99.1	99.5	99.2	99.0	100.0				
11	99.0	99.4	99.4	99.4	98.9	99.1	99.7	99.4	99.0	99.7	100.0			
12	98.9	99.3	99.3	99.3	99.0	99.0	99.6	99.3	99.0	99.7	99.9	100.0		
13	98.8	99.2	99.2	99.2	98.9	99.1	99.5	99.4	99.2	99.7	99.6	99.7	100.0	
14	98.5	99.0	99.0	99.0	98.6	98.8	99.2	99.5	99.3	99.7	99.4	99.4	99.6	100.0

Table S4. Genomic features of strains 19-857^T, 20-525, and phylogenetically-related *Mycobacterium* strains.

Species designations are as follows: 1, 19-857^T; 2, 20-525; 3, *M. arcueilense* DSM 46715^T; 4, *M. peregrinum* DSM 43271^T.

	1	2	3	4
Isolation source	sputum	sputum	water treatment and distribution system	bronchial aspiration
No. of contigs	48	53	49	78
N50 Length (bp)	386,284	377,694	374,734	201,665
Genome size (Mbp)	6.6	7.3	6.9	7.1
No. of CDS	6,292	7,004	6,712	6,672
G+C (%)	66.3	66.0	66.3	66.5
DDBJ/EMBL/GenBank accession number of draft genome	JBZDPT000000000.1	JBZDPU000000000.1	JBZTYL000000000.1	LQPP00000000.1

Table S5. Average nucleotide identity (ANI) and digital DNA–DNA hybridization (dDDH) (%) values between strains 19-857^T, 20-525 and related strains of the genus *Mycobacterium*.

1, 19-857^T; 2, 20-525; 3, *M. arcueilense* DSM 46715^T; 4, *M. peregrinum* DSM 43271^T; 5, *M. alvei* DSM 44176^T; 6, *M. septicum* DSM 44393^T; 7, *M. lutetiense* DSM 46713^T; 8, *M. setense* DSM 45070^T; 9, *M. conceptionense* JCM 15299^T; 10, *M. neworleansense* ATCC 49404^T; 11, *M. porcinum* ATCC 33776^T; 12, *M. syngnathidarum* DSM 105112^T;13, *M. houstonense* ATCC 49403^T.

ANI values >95-96% and dDDH values >70% are highlighted in bold.

strain	ANI (%)		dDDH (%)	
	1	2	1	2
1	**100.0**		**100.0**	
2	**98.8**	**100.0**	**90.1**	**100.0**
3	94.1	94.0	54.3	53.8
4	94.3	94.1	55.8	54.7
5	88.1	88.0	34.3	34.0
6	88.0	87.9	33.3	33.3
7	88.1	88.0	34.3	49.9
8	86.4	86.4	30.3	30.3
9	86.8	86.7	30.9	30.8
10	87.4	87.3	32.1	32.0
11	87.3	87.3	32.1	32.1
12	86.7	86.7	31.0	31.0
13	85.8	85.8	29.1	29.1

Table S6. Predicted isertion sequences of genomes of strains 19-857^T and 20-525.

	Isolate	seqID	family	cluster	isBegin	isEnd	isLen	strand	E-value	orfBegin	orfEnd
1	19-857^T	velvet_auto_105_1	IS1182	IS1182_60	368974	370754	1781	+	1.80E-240	369044	370262
2	19-857^T	velvet_auto_105_10	IS200/IS605	IS200/IS605_138	42317	43777	1461	+	3.00E-99	42317	43777
3	19-857^T	velvet_auto_105_15	IS3	IS3_168	62702	64033	1332	-	9.60E-117	62729	63981
4	19-857^T	velvet_auto_105_22	IS200/IS605	IS200/IS605_58	170	2248	2079	+	6.00E-19	170	2248
5	19-857^T	velvet_auto_105_23	IS110	IS110_153	71	934	864	+	1.40E-72	202	1062
6	19-857^T	velvet_auto_105_31	IS110	IS110_170	23894	25445	1552	-	7.20E-117	24280	25348
7	19-857^T	velvet_auto_105_31	IS3	IS3_406	35352	37331	1980	+	2.90E-27	35506	36884
8	19-857^T	velvet_auto_105_31	IS3	IS3_506	477005	479025	2021	-	1.80E-34	477479	478873
9	19-857^T	velvet_auto_105_31	IS21	IS21_259	524933	526795	1863	-	1.10E-11	524883	526406
10	19-857^T	velvet_auto_105_31	IS481	IS481_228	595371	596427	1057	+	7.20E-135	595468	596439
11	19-857^T	velvet_auto_105_33	IS110	IS110_140	17	851	835	-	1.60E-18	1	621
12	19-857^T	velvet_auto_105_36	IS30	IS30_173	85	1179	1095	+	3.30E-106	81	1247
13	19-857^T	velvet_auto_105_41	IS5	IS5_417	2	949	948	+	1.30E-39	1	929
14	19-857^T	velvet_auto_105_48	IS3	IS3_61	17374	18526	1153	-	1.40E-86	17711	18343
15	19-857^T	velvet_auto_105_48	IS3	IS3_369	18573	18859	287	-	1.90E-20	18626	18961
16	20-525	platanus_K_default_10	IS110	IS110_140	21595	21888	294	-	8.10E-18	21595	21888
17	20-525	platanus_K_default_14	IS21	IS21_259	363839	365701	1863	-	1.20E-11	363789	365312
18	20-525	platanus_K_default_14	IS3	IS3_374|IS3||protein:plasmid:158165	474363	475824	1462	-	1.30E-57	474779	475404
19	20-525	platanus_K_default_14	IS3	IS3_271	548169	549618	1450	-	3.40E-25	548229	549521
20	20-525	platanus_K_default_15	IS200/IS605	IS200/IS605_138	638470	639930	1461	-	2.10E-99	638470	639930
21	20-525	platanus_K_default_17	IS21	IS21_259	192176	195553	3378	-	1.50E-90	192422	195374
22	20-525	platanus_K_default_2	IS3	IS3_61	17254	18621	1368	-	4.50E-108	17694	18622
23	20-525	platanus_K_default_33	IS3	IS3_61	57	1250	1194	+	8.50E-107	1	867

24	20-525	platanus_K_default_40	IS200/IS605	IS200/IS605_96	391043	394064	3022	-	1.00E-73	391043	394064
25	20-525	platanus_K_default_40	IS110	IS110_170	706309	707804	1496	+	1.60E-151	706524	707579
26	20-525	platanus_K_default_40	IS30	IS30_173	942400	943636	1237	-	2.80E-96	942900	943673
27	20-525	platanus_K_default_42	IS5	IS5_417	3	233	231	-	1.50E-28	3	233
28	20-525	platanus_K_default_43	ISL3	ISL3_143|ISL3||protein:plasmid:23974	193	1152	960	+	6.40E-72	99	1262
29	20-525	platanus_K_default_44	IS3	IS3_271	9	1007	999	-	8.00E-96	3	1108
30	20-525	platanus_K_default_45	IS630	IS630_193	61	873	813	-	1.50E-158	1	999
31	20-525	platanus_K_default_47	IS110	IS110_153	1	1264	1264	+	1.10E-109	247	1374
32	20-525	platanus_K_default_50	IS110	IS110_208	7	569	563	-	1.20E-15	2	589
33	20-525	platanus_K_default_52	IS110	IS110_153	84	1307	1224	-	2.50E-110	2	1132
34	20-525	platanus_K_default_6	IS3	IS3_61	102923	104387	1465	-	7.60E-107	102989	104043
35	20-525	platanus_K_default_7	IS3	IS3_61	237746	239247	1502	-	3.90E-105	237819	239143

Table S7. Predicted transposons of genomes of strains 19-857^{T} and 20-525.

	Isolate	seqid	start	end	score	strand	Transposon type	Evalue
1	19-857^{T}	velvet_auto_105_31	595990	596274	153	+	gypsy	2.00E-11
2	19-857^{T}	velvet_auto_105_4	52687	53286	158	+	LINE	3.00E-12
3	19-857^{T}	velvet_auto_105_20	63983	65056	422	+	ISC1316	9.00E-47
4	19-857^{T}	velvet_auto_105_20	65062	65229	176	+	ISC1316	4.00E-16
5	19-857^{T}	velvet_auto_105_10	12814	13344	144	+	LINE	2.00E-11
6	19-857^{T}	velvet_auto_105_22	1349	2107	247	+	ISC1316	3.00E-24
7	20-525	platanus_K_default_44	6	470	121	-	gypsy	9.00E-11
8	20-525	platanus_K_default_21	68502	68903	141	-	gypsy	9.00E-11
9	20-525	platanus_K_default_45	43	915	128	-	mariner	2.00E-12
10	20-525	platanus_K_default_45	112	879	202	-	mariner_ant1	3.00E-22

11	20-525	platanus_K_default_15	638656	639891	588	-	ISC1316	3.00E-67
12	20-525	platanus_K_default_40	392729	393994	577	-	ISC1316	7.00E-66
13	20-525	platanus_K_default_40	500145	500564	156	-	LINE	1.00E-11
14	20-525	platanus_K_default_11	46970	48073	608	-	ISC1316	7.00E-71
15	20-525	platanus_K_default_22	63874	65121	598	+	ISC1316	9.00E-70
16	20-525	platanus_K_default_14	548730	549092	162	-	gypsy	2.00E-12
